# Enhanced Cytotoxicity of Aqueous *Aloe*–Silver Nanoparticles Against Human Glioma U‐87 MG Cell Line

**DOI:** 10.1049/nbt2/1937831

**Published:** 2026-06-24

**Authors:** Neha Saini, Gajanan Sonawane, Kyunghee Yun, Hyeshin Hwang, Kyungmin Kim, Bumho Yoo, Smita Zinjarde, Atul Kulkarni

**Affiliations:** ^1^ Symbiosis Centre for Nanoscience and Nanotechnology, Symbiosis International (Deemed University), Lavale, Tal. Mulshi, Pune, 412115, Maharashtra, India, ssbs.edu.in; ^2^ Alkmea Florea Private Limited, Muktiwara, Rewari, 123401, Haryana, India; ^3^ Department of Biotechnology (With Jointly Merged Institute of Bioinformatics and Biotechnology), Savitribai Phule Pune University, Ganeshkhind, Pune, 411007, Maharashtra, India, unipune.ac.in; ^4^ Bioconvergence Research Institute of HuGeX Co.,Ltd., Incheon, 22013, Republic of Korea; ^5^ Bioengineering and Medical Technologies Devision of INDSRO Defence and Aerospace Technologies Pvt., Ltd., Pune, 411041, India

**Keywords:** *Aloe barbadensis*, brain cancer, glioblastoma, green synthesis, silver nanoparticles, U-87 MG cell lines

## Abstract

The heterogeneity of glioblastoma and recurrence pose a major threat to the life of brain cancer patients. The failure of the current therapeutic regime in preventing its recurrence leads to a growing need for effective cancer treatments with lesser side effects. Previous studies have shown the potential of plants extract to synthesize silver nanoparticles (AgNPs) against U‐87 MG cell line. However, the need for optimum sized and effective nanoparticles is still not fulfilled. Hence, the current work aimed to utilize size controlled bioavailable AgNPs capped with *Aloe barbadensis* (aqueous extracts) and study their effectiveness against U‐87 MG cells. Two different concentrations of *Aloe vera* extract (ALE) viz. ALE1 and ALE2, were used. Their physiochemistry was characterized via UV–Vis, dynamic light scattering (DLS), FE‐SEM (50–60 nm diameter), and energy dispersive X‐ray (EDX). The observed zeta potentials were −8 and −16 mV for ALE1– and ALE2–AgNPs, respectively. This indicated that ALE2–AgNPs were more stable. Further, the anticancer potency of both ALE–AgNPs at 0, 24, and 48 h was evaluated. MTT‐assay revealed ALE2–AgNPs were more cytotoxic, significantly inhibiting the proliferation of U‐87 MG, indicating their dose‐dependent cytotoxicity. At an effective concentration of ALE2–AgNPs, (i.e., 10 µg/mL) viability of cancer cell line decreased significantly to 40% after 24 h of treatment. In apoptotic analysis pronounced elevation in early apoptotic cells was noted (5.1% and 9.9% for ALE1– and ALE2–AgNPs, respectively). The significant cytotoxicity and observed apoptosis induction shows ALE–AgNPs’s therapeutic potential against glioblastoma and aqueous extract shows its high biocompatibility, thus having significant probability to be translated in clinical settings.

**Summary**

• Use of aqueous extracted and biocompatible *Aloe barbadensis* silver nanoparticles (AgNPs; *Aloe vera* extract [ALE]–AgNPs).
• Cytotoxic studies were carried out on U‐87 MG cell lines.
• ALE–AgNPs dose and time dependent cytotoxic effect is prominent.
• Effective wound healing is observed in 48 h.

## 1. Introduction

Cancer continues to pose major treatment challenges [1–3]. The heterogeneity of glioblastoma [4] and failure of current therapeutic regime due to low drug uptake because of ABC efflux transporters present in blood–brain barrier [4] fails the current regimes. Glioblastomas are the most common form of human glioma that are difficult to treat [4–9]. The main reasons for this are frequent recurrences are resistance of conventional drugs [10, 11], their side‐effects during and after treatment schedules. Conventional treatment regimens have limitations, due to low specificity, selectivity, and systemic side‐effects [2, 12]. Moreover, contemporary treatments involving surgical interventions and chemotherapy are highly invasive [13–15]. Although radiation therapy is preferred on account of its noninvasive nature, it poses severe after‐therapy effects [6, 14, 16]. Further, traditional treatments often result in severe side effects, highlighting the demand for alternative therapies.

In the backdrop of this scenario, the potential of nanotechnology for cancer therapeutics [2, 17] has been explored. Nanoparticle‐based medicines are emerging as promising alternatives to conventional glioblastoma treatment regimens [15, 18, 19]. Green synthesized ones are especially significant in this regard [1, 2, 20, 21]. Distinct physicochemical specialties of nanoparticles help them to interact with cells at the molecular level [2]. Many studies have shown that silver nanoparticles (AgNPs) exhibit anticancer potency [22] against various cell lines including U‐87 MG [23–25]. This is mainly due to the generation of silver ions [15, 26, 27]. Further, literature review reveals that acidic pH surrounding cancer cells cause more silver ions to release in leading to increased cytotoxicity [15, 28]. These also trigger apoptosis and prevent cancer metastasis [2].

Past studies have reported the use of plant callus of various plants [29–31], rhizomes and stems of *Alocasia odora*, cauliflower to green synthesize AgNPs [20, 32]. These AgNPs were examined for dose‐dependent cytotoxic effects [33, 34] on human U‐87 MG cell lines and used as anticancer agents. It was noted that AgNPs synthesized by using stems at a concentration of 43.40 µg/mL were most cytotoxic [35]. AgNPs formed by using *Citrus aurantium* [6] and fruit of *Diospyros malabarica* were also effective [36]. In other studies, *Zinnia elegans* ethanolic extract derived AgNPs have shown anticancer effect [15]. Further, AgNPs synthesized by using extracts of *Potentilla fulgens* and *Lavandula angustifolia* had IC_50_ values of 45–75 and 7.536 µg/mL, respectively [24, 37]. AgNPs obtained from tuber extracts of *Pueraria tuberosa* exhibited an IC_50_ value of 6.053 µg/mL against U‐87 MG cell lines and *Plumeria alba* mediated AgNPs showed IC_50_ of 9.77 µg/mL for on U‐118 MG cells [38, 39].

Some components associated with *Aloe barbadensis* are also reviewed to demonstrate bioactive properties [40]. *Aloe*‐emodin was found to have therapeutic applicability against gastric cancer cells [41], mediate gene modulation in U‐87 MG [7] and facilitate dose and time dependent inhibition of U‐87 MG proliferation [34, 42]. These flavonoids were also seen to induce apoptosis and exhibit inhibitory effect on glioblastoma cell lines [8, 9, 42, 43].

*Aloe vera* has also been able to mediate green synthesis of AgNPs [36]. Such nanoparticles offered benefits associated with both *Aloe vera* and silver [7–9, 21, 34]. While silver contributed towards antimicrobial, anticancer, and cytotoxic properties [24], *Aloe vera* aided in reducing side effects on surrounding cells [8, 9, 44]. Similarly, other nanoparticles like cesium oxide, green synthesized (using *Aloe*) also demonstrated cytotoxic effects on U‐87 MG cell lines [45]. There has also been emphasis on evaluation of cytotoxic effect of phytochemicals [46]. The cytotoxicity of cesium nanoparticles was seen to be enhanced in the presence of a phenolic compound—Urolithin B, showing the feasibility of drug‐nanoparticle conjugate in glioblastoma management [47]. Moreover, the hydrogels formed via *Aloe* impeded the growth of tumor spheroids as well [48]. Another study, *Aloe*‐emodin was seen to regulate cell growth via inducing apoptosis on U‐87 MG cells, in a time‐ and dose‐dependent manner [34].

Even though, stable glioblastoma cell lines are rather more homogenous than in vivo cells and have various subtypes too. Further, the primary U‐87 MG cultures are difficult to establish due to senescence at very early stage, spontaneous or idiopathic apoptosis, mitotic catastrophe, and so on. Thus, to prevent the lack of standardization and to establish comparison efficiency, an optimized culture (i.e., adherent culture) method has been opted to prolong its in vitro maintenance with preserved phenotype and genotype [49].

The efficacy of nanoparticles is known to depend on their uptake by cell lines that in turn is determined by factors such as size, surface type of nanoparticles and type of cell lines being studied [2]. Collectively, from these observations we concluded that the use of natural *Aloe* extract to prepare AgNPs against U‐87 MG cell line would have more potential as shown in Table 1. However, when compared with chemotherapeutic drug like temozolomide (TMZ), the previous studies shows TMZ is required in higher concentrations of 500 µM to kill the cells and remain highly cytostatic till 6 days [50]. The various extracts used to synthesize AgNPs have shown good IC_50_ values, however, some have sizes ranging more than 100 nm that can possibly pose hurdle in their passage via blood–brain barrier. Thus, to facilitate cytotoxicity against U‐87 MG via *Aloe vera* extract (ALE)‐AgNPs, factors like size, surface charge, concentration and incubation time were optimized in the present study. Hence, the prime impetus of our study was green synthesis aqueous with natural *Aloe* extract as reductant and as an agent for keeping the surface significantly negative so that easy uptake by U‐87 MG glioblastoma cell lines could be ensured. Further, the dose and time dependent activity of ALE–AgNPs were evaluated via two different types of as‐synthesized ALE–AgNPs. As compared to alcoholic nanoparticles, aqueous nanoparticles are better in terms of biocompatibility, and higher efficacy towards glioblastomas with sevenfolds enhanced cytotoxicity. The aqueous ALE–AgNPs synthesized using green approach clearly demonstrated cytotoxic and apoptotic activity against U‐87 MG glioblastoma cell lines (observed during MTT assay; apoptotic activity and scratch assay) [51]. This study like another investigation [13] highlights the importance of phytochemicals and green synthesis routes in cancer therapeutics.

**Table 1:** Comparative chart for IC_50_ values of various extracts to synthesize AgNPs.

IC_50_ at 24 h (µg/mL)	Size (nm)	References
10–12	Less than 60 nm	Current study
43.40–75	—	[24, 34–36]
7.536	~100	[37]
6.053	162.72 ± 5.02	[38]
0.94 (ethanolic)	—	[15]

## 2. Materials and Methods

### 2.1. Chemicals, Media, and Media Components

Silver nitrate (AgNO_3_), dimethyl sulfoxide (DMSO), 3‐(4,5‐dimethylthiazol‐2‐yl)‐2,5‐diphenyltetrazolium bromide (MTT), trypsin, propidium iodide (PI), Dulbecco’s modified Eagle’s medium (DMEM), fetal bovine serum (FBS), and trypsin phosphate versene glucose (TPVG) were procured from Sigma–Aldrich Chemicals. The studies have been performed using Milli‐Q‐water (18.2 MΩ·cm).

### 2.2. Preparation of Crude ALE Powder

Leaves of *Aloe barbadensis* (*Aloe vera*) were collected from the campus of Symbiosis International (deemed University), India. They were thoroughly washed with distilled water, sun dried the whole leaves with gel and green cover, weighed, and grounded [44]. The obtained powder was stored in airtight containers away from moisture and light at ambient temperature.

### 2.3. Characterization of Optimized Nanoparticles

**Figure 1:**
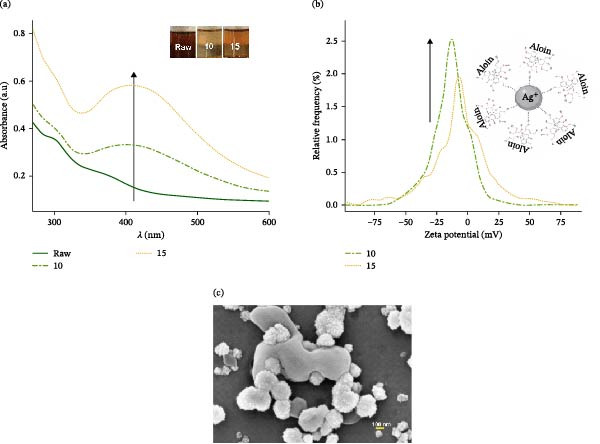
Surface and morphological characterization of ALE AgNPs. (a) UV–visible absorbance spectra of raw extract, ALE1–AgNPs and ALE2–AgNPs. Inset shows visual observations of raw extract, reaction mixtures containing 10% and 15% ALE (tubes marked 10 [ALE1] and 15 [ALE1], respectively). (b) Zeta potential of ALE1–AgNPs and ALE2–AgNPs. Inset shows a pictorial representation of the *Aloe*‐nanoparticle structural association. (c) Representative FE‐SEM image of ALE AgNPs (yellow scale at bar equivalence = 100 nm).

ALE mediated AgNPs were synthesized via top‐down approach [1, 52]. A previously published protocol was used [53]. In brief, 5 mM AgNO_3_ (42.5 mg) was added in beakers containing 50 mL of preheated distilled water. For synthesizing ALE1– and ALE2–AgNPs, 1 mL of ALE (that contains 10 mg *Aloe* powder per mL of DMSO and 15 mg *Aloe* powder per mL of DMSO, respectively) was added in a dropwise manner in different beakers containing 50 mL of 5 mM AgNO_3_ and incubated (85°C, 550 rpm, 72 h). As reported earlier, ALE acted as the reducing and capping agent [44]. UV–visible spectroscope (Jasco V‐750, Made in Japan) revealed an absorption peak at 420 nm that is characteristic of AgNPs as shown in Figure 1a. The hydrodynamic diameters of ALE AgNPs were determined via dynamic light scattering (DLS) technique on Anton Paar equipment (Model: BM10, Make: Litesizer500, Made in Germany) as reported earlier [54]. The structural morphology of the AgNPs were observed via FESEM.

### 2.4. Antitumor Efficiency

#### 2.4.1. In Vitro Cultivation of Cell Lines

U‐87 MG glioblastoma (astrocytoma) cell line (Passage Number p43) was procured from National Centre for Cell Sciences, (NCCS), Pune, India. The cell line was maintained in Minimum essential Eagle’s Medium (MEM; 90%) and FBS (10%). The cell lines were kept at 37°C and 5% CO_2_ for incubation [2].

### 2.5. MTT Assay

Cytotoxicity of ALE–AgNPs on the proliferation/cell viability of U‐87 MG cancer cell lines, were evaluated via MTT assay [2, 23]. 20,000 cells/100 µL/well were seeded in 96‐well plates and kept in incubator for 24 h to facilitate adhesion. These cells were individually incubated further with ALE1– and ALE2–AgNPs. The viability assay was performed as per the literature [14, 15, 35, 36]. The cell lines (U‐87 MG) were incubated with AgNPs for 24 and 48 h before cell viability assay using MTT reagents was carried out. Untreated cells were considered as control samples. After incubation, 100 μL of MTT solution (0.5 mg/mL of PBS) was added to each well and kept in CO_2_ incubator at 37°C for 4 h. The 100 μL DMSO was loaded to dissolve formazan crystals, and the optical density (OD) was measured by using an ELISA reader at 570 nm. The mean OD from triplicate samples was determined and cytotoxicity was calculated via IC_50_ values indicating the concentration of ALE–AgNPs that were required to inhibit 50% cell growth of U‐87 MG.

## 3. Results and Discussion

### 3.1. Characterization of ALE–AgNPs

As per a previous report [44], synthesis of ALE–AgNPs was confirmed by a change in color of the solution as depicted in the inset of Figure 1a. This indicated reduction of silver ions (Ag^+^) to Ag^0^ [52]. UV–visible spectroscopy revealed an absorption peak at 420 nm that is characteristic of AgNPs (Figure 1a). DLS analysis revealed the zeta potential analysis indicated a value of −8 mV for ALE1–AgNPS and −16 mV ALE2–AgNPS, respectively, suggesting good stability as shown in Figure 1b. AgNPs with size less than 60 nm are considered suitable for biological applications [55]. Morphology of ALE2–AgNPS showing surface characteristics and size are presented in Figure 1c. This image shows that the nanoparticles were 60 ± 5 nm in diameter. Energy dispersive X‐ray (EDX) spectroscopic analysis of these nanoparticles confirmed the presence of elemental silver and magnesium too (Figures S1 and S2). Figure S1 shows the EDX % of Mg^2+^ (7.49) and Ag^+^ (44.05) for ALE1 and Figure S2 shows the EDX % of Mg^2+^ (14.90) and Ag^+^ (24.29) for ALE2.

### 3.2. Cytotoxicity Assessment of ALE–AgNPs via MTT Assays

**Figure 2:**
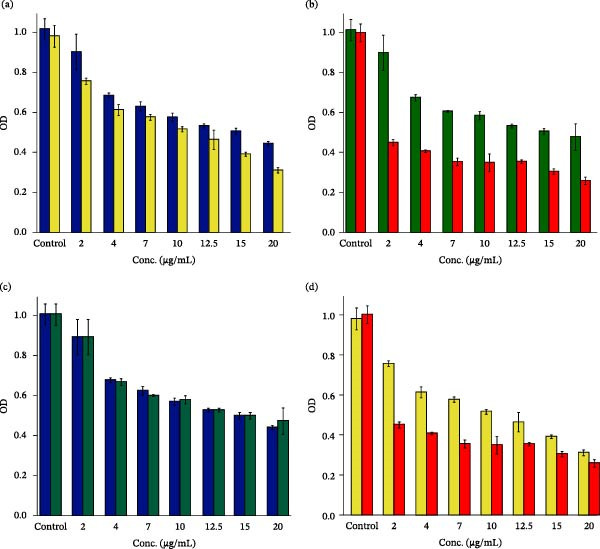
Time and concentration dependent cytotoxicity of ALE AgNPs on U‐87 MG glioblastoma cell lines. (a) Impact of ALE1–AgNPs at 24 and 48 h (blue and yellow bars, respectively). (b) Impact of ALE2–AgNPs at 24 and 48 h (green and red bars, respectively). (c) Impact of ALE1–AgNPs and ALE2–AgNPS at 24 h (blue and green bars, respectively). (d) Impact of ALE1–AgNPs and ALE2–AgNPS AgNPs at 48 h (yellow and red bars, respectively).

Data related to the cytotoxic impact of ALE‐AgNPs on proliferation of U‐87 MG cell lines assessed via MTT assay is presented in Figure 2. MTT assay results demonstrated that ALE–AgNPs have a dose‐dependent cytotoxic effect as also noted previously with U‐87 MG glioblastoma cell lines [34]. For % cell viability of U‐87 MG glioblastoma cell lines at different time intervals with various concentration of *Aloe*–AgNPs refer to Figure S3. Reduction in cell viability was significant at higher concentrations of ALE–AgNPs (20 µg/mL) as compared to 10 µg/mL, suggesting their potential as an effective antitumor agent. After treatment with 10 µg/mL of ALE1–AgNPs after 24 h, cell viability decreased by 40%. At higher concentrations (20 µg/mL), cell viability further decreased by 10%, indicating the cytotoxic nature of the nanoparticles (Figure 2a).

The observed decrease in OD with increasing concentration of nanoparticles, indicated dose dependent cytotoxic effect. Time‐dependent increase in cytotoxicity was also observed suggesting that prolonged exposure to ALE–AgNPs enhanced their antitumor efficacy. The negative zeta potential of the nanoparticles presumably facilitated uptake by glioblastoma cells and contributed to the observed cytotoxic effects.

ALE2–AgNPs showed significantly lower IC_50_ values (Figure 2b) compared to ALE1–AgNPs indicating better cytotoxicity of the former type of nanoparticles. The impact of other cofounding variables such as incubation time, concentration and dosage on this assay via U‐87 MG cell lines was also evaluated. These results also indicated that the cytotoxic effect with a significantly low cell viability after 48 than 24 h treatment for both ALE1– and ALE2–AgNPs (Figure 2c,d).

### 3.3. Cell Migration in Scratch Assay

**Figure 3:**
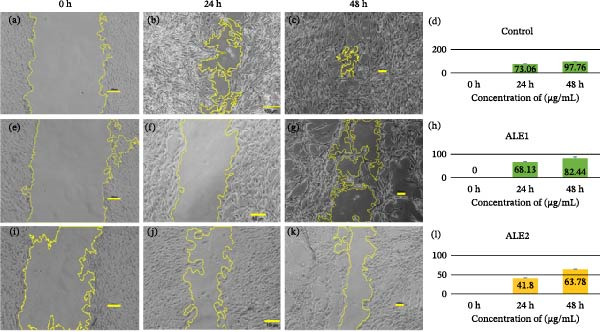
Wound healing assay and cytotoxicity assessment of U‐87 MG cells treated with media (control), ALE1–AgNPs, and ALE2–AgNPs. Representative images showing wound healing progress (closure) on treatment with (a)–(c) control without AgNPs at 0, 24, and 48 h respectively, (e)–(g) ALE1–AgNPs treated at 0, 24, and 48 h, respectively, (i)–(k) ALE2–AgNPs treated at 0, 24, and 48 h, respectively. The assay evaluates the cytotoxic effects of Control, ALE1, and ALE2–AgNPs on cell migration and viability following treatment after different time intervals as shown in Subparts (d), (h), and (l), respectively.

ALE–AgNPs has different impact on the cell’s migration in U‐87 MG glioblastoma cell lines over (0, 24, and 48 h) asana evaluated via scratch assays [6]. Scratch assay images depicting wound healing time and progress of wound healing are shown in Figure 3. In control groups (Figure 3a–d) consistent and gradual wound closure was observed, indicative of normal cell migration and proliferation [56, 57] in absence of ALE–AgNPs treatment (Figure 3a–d). Conversely, in treatment groups, a stark variation in the progress of wound healing was observed (Figure 3e–l and Table 2).

**Table 2:** Depiction of wound closure rate/speed and wound closure % of untreated and ALE1‐ and ALE2‐treated U‐87 MG cell line.

Experimental group	Initial area (*A*_i_, µm^2^) *t* = 0	Area (µm^2^) *t* = 24 h	Final area (*A*_f_, µm^2^) *t* = 48 h	Wound closure % (*A*_i_ − *A*_f_/*A*_i_) × 100	Initial width (*W*_i_, µm at *t* = 0)	Final width (*W*_f_, µm at *t* = 48 h)	Migration rate (*W*_i_ − *W*_f_/t) (µm/h)
Control	527,942	139,268	13,073.4	97.524	200	340	7.085
ALE1	514,241	158,717	74,840.4	85.446	270	280	5.833
ALE2	509,977	289,953	176,516	65.387	330	190	3.958

Cells treated with ALE1–AgNPs (Figure 3e–h and Table 2), exhibited a noticeable delay in wound closure at 24 h, with significant impedance even at 48 h, suggesting effective suppression of cell migration. This impact was more pronounced in the group treated with ALE2–AgNPs (Figure 3i–l and Table 2). Further, the minimal wound closure was evident after 24 and 48 h, highlighting drastic impairment in cell migration. These findings underscore the function of ALE–AgNPs in hindering glioblastoma cell migration in a dose‐dependent manner as also observed previously [34].

### 3.4. Proposed Mechanism Underlying Anticancer Activity of ALE–AgNPs

Previous studies have shown that AgNPs exhibit anticancer potential [1]. This effect is generally via generation of silver ions and superoxide radicals [15, 58–60]. Furthermore, literature indicates that with chemically and green synthesized AgNPs, more silver ions are released in the acidic surrounding of cancer cells thereby contributing towards increased cytotoxicity. Due to leaky tumor vasculature, upregulated metabolism leads to increased uptake of AgNPs by cancer cells via enhanced permeability and retention (EPR) effect [15].

### 3.5. Flow Cytometric Analysis

**Figure 4:**
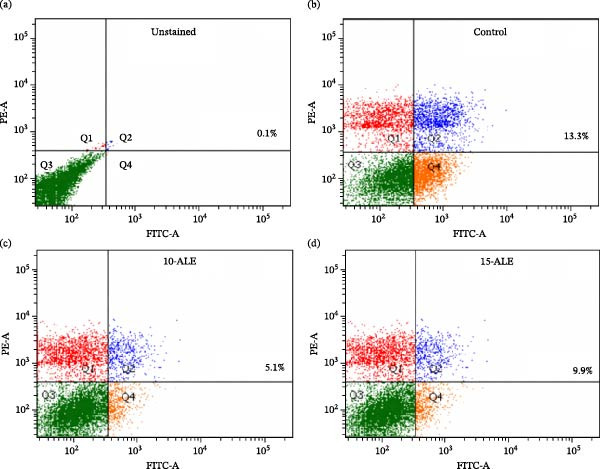
Apoptosis assay via Annexin V‐FITC after 24 h incubation: (a) Unstained: negative control. (b) Stained, untreated: positive control. (c) Treated with ALE1–AgNPs. (d) Treated with ALE2–AgNPs cells. An enhanced population of early apoptotic cells treated with ALE2–AgNPs was observed.

The apoptosis assay was performed via Annexin V‐FITC staining [61] on U‐87 MG glioblastoma cell lines treated and incubated for 24 h with ALE1– and ALE2–AgNPs using flow cytometry. The assay was performed on four distinct groups as depicted in Figure 4.

The untreated group in Figure 4a acted as the baseline to assess the natural apoptotic rate within the U‐87 MG cell population (untreated negative control). In Figure 4b, treated positive control sample shows a significant shift in cell population towards the early apoptotic phase, indicating the effectiveness of the treatment protocol. Remarkably, in cells treated with ALE1–AgNPs (Figure 4c), a marked increase in late apoptotic cells (5.1%) was observe. This indicated the ability of nanoparticles to induce apoptosis like observed by other investigators [8, 9, 13]. Furthermore, the group treated with ALE2–AgNPs (Figure 4d) showed an even more pronounced elevation of 9.9% in early apoptotic cells, underscoring a dose‐dependent relationship [34]. The results show the potent apoptotic effect of ALE–AgNPs on U‐87 MG cell lines, with higher concentrations leading to a substantial rise in early apoptotic cell populations suggesting their promising antitumor potential as also observed previously [43].

## 4. Discussion

Advancements in nanoscience research have an unparalleled impact on the creation of next‐generation therapeutics, diagnostics, and devices for challenging diseases including brain cancer. Nanomedicine has boosted targeted medication delivery, minimized side effects, improved biocompatibility, increased drug solubility, and augmented therapeutic efficacy [62, 63]. The aqueous ALE–AgNPs synthesized [64] using green approach clearly demonstrated cytotoxic and apoptotic activity against U‐87 MG glioblastoma cell lines. The concentration dependent action of ALE2–AgNPs along with the time dependent cytotoxicity (observed during MTT assay) and apoptotic activity indicates the crucial impact of concentration on cytotoxicity as noted on a previous occasion as well [51]. This study like another investigation [13] highlights the importance of phytochemicals and green synthesis routes in cancer therapeutics. The potent cytotoxicity and migration inhibition by higher concentrations of ALE–AgNPs affirm their potential as a therapeutic strategy for targeting glioblastoma. Establishment of ALE–AgNPs as a therapeutic agent would help to reduce brain cancer recurrence, and therapeutic comorbidities. *Aloe*–silver as onco‐therapeutic agent would be useful in glioblastoma recurrence management as evident through results of the scratch assay shown in Figure 3. Various other nanomaterials have already been evaluated for their antitumor effects. This study is evidence that certain phytochemicals are better alternative to the chemical drugs which leads to stringent comorbidities. *Aloe* was used for its cooling and antimicrobial effects. Silver has already been proven to have antitumor and anticancer effects. Further, the concentration dependent impact was also evident through all the in vitro studies including MTT, wound healing and apoptosis assays that were performed. The apoptosis assay as shown in Figure 4 made it evident that ALE1– and ALE2–AgNPs induced apoptosis in 5.1% and 9.9% of cells, respectively. MTT assay was utilized to test the metabolic activity, dosage toxicity, and cellular viability on U‐87 MG cell‐line of human glioblastoma. We evaluated the significant impact of potential cofounding variables like incubation time (at 0, 24, and 48 h), and dosage concentration/treatment toxicity, released cell lysate and extrusion of formazan crystals on this assay via U‐87 MG cell lines (with 10 µL of 10 mg/mL MTT–ALE–AgNPs solution serial dilution). Furthermore, this research outcome will lead to determine therapeutic range of ALE–AgNPs that are effective at ultralow concentrations. Alongside, via scratch assays, cell migration was seen to be lower in ALE2 treated cell lines as compared to ALE1 and control samples, as clearly stated in Table 2. This depicted the feasibility of the nanoparticles against recurrence of glioblastoma. However, further validation of the effectiveness of ALE–AgNPs is in vivo systems is warranted to evaluate their feasibility in clinical tests. The distinct biocompatibility of ALE–AgNPs, their eco‐friendly synthesis and easy preparation make it a suitable therapeutic agent against glioblastoma. However, the heterogeneity of glioblastoma, the test results might vary as per the stages of curation (passage number) and the concentration of reductant used during synthesis (as already proven in this study). Thus, creating a panel of nanomaterials would provide better biocompatibility and higher cytotoxicity.

## 5. Conclusion

This study highlights the potential of *Aloe*‐mediated AgNPs as promising antitumor agents for glioblastoma treatment. The green synthesis approach provides a biocompatible and environmentally friendly method for producing AgNPs. The characterized ALE–AgNPs showed significant cytotoxic effects on U‐87 MG glioblastoma cell lines indicating their potential for further development as a therapeutic agent. Further, we got a 6–7‐folds enhancement in cytotoxicity against U‐87 MG cell line with IC_50_ value of 10–12 µg/mL with ALE–AgNPs as compared with other green extracts as studied by other researchers.

### 5.1. Limitations of the Study

The present study can be further extended using different solvents with varying pH ranges and extended concentrations. However, due to resource‐limitedness, financial constraints, and the exploratory nature of the experimentations, the cytotoxicity assays and characterization analysis was limited to optimized concentrations and certain basic tools.

## Author Contributions

**Neha Saini**: writing – original draft, writing – review and editing, methodology, experimentation, investigation, formal analysis, data curation, conceptualization, software, validation, visualization. **Gajanan Sonawane**: writing – review and editing, experimentation, investigation, formal analysis. **Kyunghee Yun, Hyeshin Hwang, Kyungmin Kim, and Bumho Yoo**: project administration, supervision, funding acquisition. **Smita Zinjarde**: resources, project administration, supervision, writing – review and editing. **Atul Kulkarni**: conceptualization, resources, project administration, supervision, writing – review and editing.

## Funding

No funding was received for this manuscript.

## Conflicts of Interest

The authors declare no conflicts of interest.

## Supporting Information

Additional supporting information can be found online in the Supporting Information section.

## Supporting information


**Supporting Information** Figure S1. Energy‐dispersive X‐ray spectroscopy (EDX) spectrum of *Aloe vera*–mediated *Aloe*–AgNPs using 10 mg ALE1, showing that the nanoparticles contained elements of silver and phytochemical constituents. The characteristic peaks of silver nanoparticles are visible, and the other peaks are attributed to the elements of the phytochemical capping and stabilizing agents. Figure S2. The energy‐dispersive X‐ray spectroscopy (EDX) spectrum of the *Aloe vera*–mediated *Aloe*–AgNPs synthesized using 15 mg ALE2 shows the presence of the elements and successful incorporation of silver nanoparticles. The results from the analysis confirms the presence of silver as the major constituent and the presence of successful green synthesis and stabilization of nanoparticles by biomolecules of *Aloe*. Figure S3. Cell viability of U‐87‐MG glioblastoma cells after exposure of *Aloe vera*–mediated *Aloe*–AgNPs for different concentrations and incubation time: The MTT assay was used to evaluate cell viability, revealing that the cytotoxic activity of *Aloe*–AgNPs is concentration‐ and time‐dependent on glioblastoma cells. The results show that the cell viability gradually decreased with increasing concentrations of nanoparticles and exposure time, indicating better anticancer effect of *Aloe*–AgNPs on glioblastoma cells at 48 h.

## Data Availability

The relevant data have already been included in the manuscript while the supporting studies and results are provided in the supporting file.
