# Supplementary material for: Enhanced Cytotoxicity of Aqueous Aloe–Silver Nanoparticles Against Human Glioma U‐87 MG Cell Line
Source: IET Nanobiotechnol. 2026 Jun 24;2026:1937831. doi: 10.1049/nbt2/1937831 (PMC13291803; doi:10.1049/nbt2/1937831)
Supplement: Supplementary file 1 — Supporting Information Figure S1. Energy‐dispersive X‐ray spectroscopy (EDX) spectrum of Aloe vera–mediated Aloe–AgNPs using 10 mg ALE1, showing that the nanoparticles contained elements of silver and phytochemical constituents. The characteristic peaks of silver nanoparticles are visible, and the other peaks are attributed to the elements of the phytochemical capping and stabilizing agents. Figure S2. The energy‐dispersive X‐ray spectroscopy (EDX) spectrum of the Aloe vera–mediated Aloe–AgNPs synthesized using 15 mg ALE2 shows the presence of the elements and successful incorporation of silver nanoparticles. The results from the analysis confirms the presence of silver as the major constituent and the presence of successful green synthesis and stabilization of nanoparticles by biomolecules of Aloe. Figure S3. Cell viability of U‐87‐MG glioblastoma cells after exposure of Aloe vera–mediated Aloe–AgNPs for different concentrations and incubation time: The MTT assay was used to evaluate cell viability, revealing that the cytotoxic activity of Aloe–AgNPs is concentration‐ and time‐dependent on glioblastoma cells. The results show that the cell viability gradually decreased with increasing concentrations of nanoparticles and exposure time, indicating better anticancer effect of Aloe–AgNPs on glioblastoma cells at 48 h. [file NBT2-2026-1937831-s001.docx]

**Supplementary Figures**

**S Figure 1. EDX (ALE1 10 mg)**


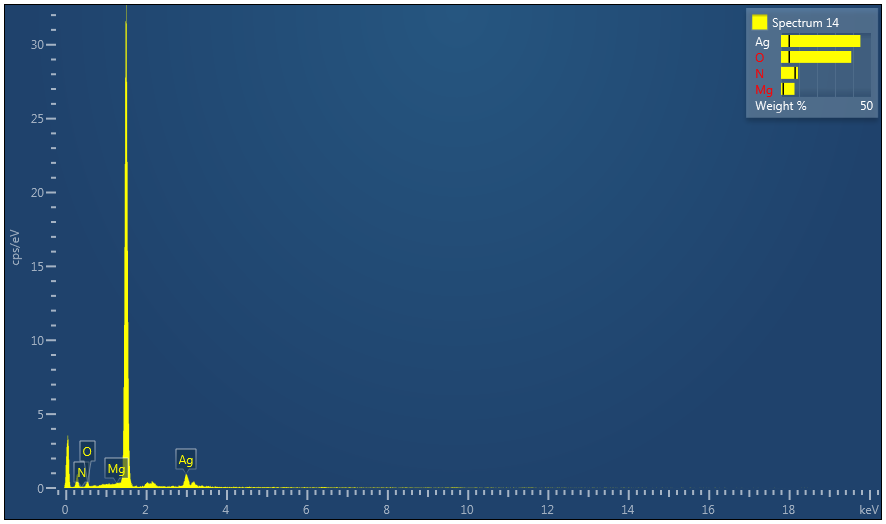


| Element | Line Type | Apparent Concentration | k Ratio | Wt% | Wt% Sigma | Standard Label | Factory Standard | Standard Calibration Date |
| --- | --- | --- | --- | --- | --- | --- | --- | --- |
| N | K series | 0.79 | 0.00141 | 9.43 | 7.83 | BN | Yes |  |
| O | K series | 1.56 | 0.00525 | 39.02 | 4.60 | SiO2 | Yes |  |
| Mg | K series | 0.52 | 0.00346 | 7.49 | 1.24 | MgO | Yes |  |
| Ag | L series | 4.37 | 0.04368 | 44.05 | 4.59 | Ag | Yes |  |
| Total: |  |  |  | 100.00 |  |  |  |  |

**S Figure 2. EDX (ALE 2 – 15 mg)**


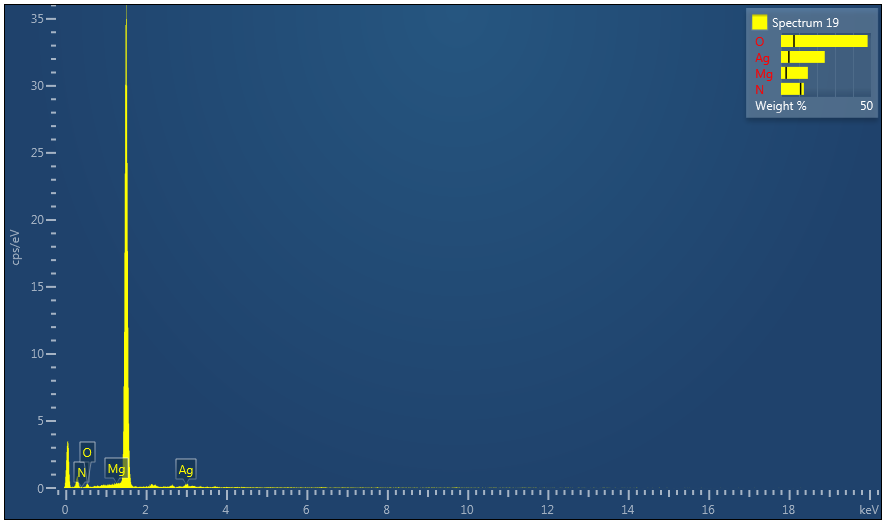


| Element | Line Type | Apparent Concentration | k Ratio | Wt% | Wt% Sigma | Standard Label | Factory Standard | Standard Calibration Date |
| --- | --- | --- | --- | --- | --- | --- | --- | --- |
| N | K series | 0.65 | 0.00116 | 12.72 | 10.86 | BN | Yes |  |
| O | K series | 1.13 | 0.00380 | 48.08 | 7.21 | SiO2 | Yes |  |
| Mg | K series | 0.52 | 0.00343 | 14.90 | 2.84 | MgO | Yes |  |
| Ag | L series | 1.06 | 0.01065 | 24.29 | 4.38 | Ag | Yes |  |
| Total: |  |  |  | 100.00 |  |  |  |  |

**S Figure 3.**








**S Figure 3:** % cell viability of U87 MG glioblastoma Cell lines at different time intervals with

various concentration of Aloe-AgNPs
